# Myocardial Infarction Complications After Surgery in Vietnam: Estimates of Incremental Cost, Readmission Risk, and Length of Hospital Stay

**DOI:** 10.3389/fpubh.2021.799529

**Published:** 2021-12-10

**Authors:** My Hanh Bui, Quynh Long Khuong, Phuoc Thang Dao, Cao Phuong Duy Le, The Anh Nguyen, Binh Giang Tran, Duc Hung Duong, Tuan Duc Duong, Tien Hung Tran, Hoang Ha Pham, Xuan Thanh Dao, Quang Cuong Le

**Affiliations:** ^1^Department of Tuberculosis and Lung Diseases, Hanoi Medical University, Hanoi, Vietnam; ^2^Department of Functional Exploration, Hanoi Medical University Hospital, Hanoi, Vietnam; ^3^Center for Population Health Science, Hanoi University of Public Health, Hanoi, Vietnam; ^4^Department of Monitoring and Evaluation, Interactive and Research Development, Ho Chi Minh City, Vietnam; ^5^Department of Interventional Cardiology, Nguyen Tri Phuong Hospital, Ho Chi Minh City, Vietnam; ^6^Department of Intensive Care, Huu Nghi Hospital, Hanoi, Vietnam; ^7^Department of Gastroenterology Surgery, Viet Duc Hospital, Hanoi, Vietnam; ^8^Department of Cardiovascular Surgery, Bach Mai Hospital, Hanoi, Vietnam; ^9^Vietnam Social Insurance, Hanoi, Vietnam; ^10^Department of Orthopedic, Hanoi Medical University Hospital, Hanoi, Vietnam; ^11^Department of Neurology, Hanoi Medical University, Hanoi, Vietnam

**Keywords:** coronary artery disease, perioperative, economic burden, re-hospitalization, length of stay

## Abstract

Myocardial infarction is a considerable burden on public health. However, there is a lack of information about its economic impact on both the individual and national levels. This study aims to estimate the incremental cost, readmission risk, and length of hospital stay due to myocardial infarction as a post-operative complication. We used data from a standardized national system managed by the Vietnam Social Insurance database. The original sample size was 1,241,893 surgical patients who had undergone one of seven types of surgery. A propensity score matching method was applied to create a matched sample for cost analysis. A generalized linear model was used to estimate direct treatment costs, the length of stay, and the effect of the complication on the readmission of surgical patients. Myocardial infarction occurs most frequently after vascular surgery. Patients with a myocardial infarction complication were more likely to experience readmission within 30 and 90 days, with an OR of 3.45 (95%CI: 2.92–4.08) and 4.39 (95%CI: 3.78–5.10), respectively. The increments of total costs at 30 and 90 days due to post-operative myocardial infarction were 4,490.9 USD (95%CI: 3882.3–5099.5) and 4,724.6 USD (95%CI: 4111.5–5337.8) per case, while the increases in length of stay were 4.9 (95%CI: 3.6–6.2) and 5.7 (95%CI: 4.2–7.2) per case, respectively. Perioperative myocardial infarction contributes significantly to medical costs for the individual and the national economy. Patients with perioperative myocardial infarction are more likely to be readmitted and face a longer treatment duration.

## Introduction

Myocardial infarction (MI) is a common cause of coronary artery disease (CAD) and is a cause of quality-of-life decline and in-hospital mortality (1). MI occurs when the blood flow that supplies oxygen to the myocardium is reduced, leading to fatal myocardial ischemia and necrosis (2, 3). The incidence of MI complications during and after surgery ranges from 0.3 to 33%, according to age group, patient cardiac history, and type of intervention (4–6), while myocardial injury rates are from 9.9 to 17.9% (7). Perioperative myocardial infarction (PMI) occurs most frequently within 1–3 days of surgery, with 57.4% reported 1 day after surgery and nearly 20% more by the third day (8, 9). Although the mortality rate for PMI is on a downward trend (10, 11), in-hospital numbers was 11.6% among patients who are 45 years old or older in 2011 and reached 75.6% over the period 2003–2015 (12, 13). Further, Dennis et al. recorded a rate of 30-day post-discharge mortality at 4.4% among patients who are 65 years old or older in 2017 (10). Alongside the risk of immediate onset, PMI might occur asymptomatically, and so close post-operative follow-up and periodical clinical evaluation is needed.

PMI is not only life-threatening to patients but also results in long-term loss of quality of life. A systematic review indicated the 30-day readmission rate at 12% and was found to be up to 19.1% by Smilowitz (14, 15). Umesh et al. showed a high risk of readmission due to PMI at least once within 12 months of discharge, at 21.3% (16). A higher possibility of admission could lead to longer treatment duration, as has been shown by the correlation between MI and the longest length of stay (11). The burden of treatment cost has been highlighted in a number of studies (17–19), and hospitalization costs increased considerably among patients who undergo intervention (17), with an accumulated high burden on the national economy (19). The cost of MI treatment cost in Vietnam in 2013 was estimated to be US$ 2,503 per hospitalization, which was significantly higher than contemporary GDP per capita (20).

Vietnam is a low-middle income country (LMIC) locating in the South-East region of Asia, with an estimated population in 2019 of ~96.5 million people (21). Over the same period of 2019, Vietnam's GDP showed an increase of 7.02%, and per capita income was reported at 4.3 million dong (21). Even though treatment therapies for CHD are constantly evolving, CHD negatively contributed 13.22% of all-cause mortality in 2018 (22). Hoa et al. estimated that the prevalence of ST-segment elevation MI in patients who had undergone percutaneous coronary therapy was 14.5% (23). Nonetheless, little is known about the burden of PMI in terms of the risk of readmission, length of hospital stay (LOS), or burden of hospitalization costs. Additional studies are needed to understand the burden of PMI in LMICs and to provide valuable findings to promote protective strategies against impoverishing expenditure as part of the Global Surgery 2030 (24).

This study was conducted to explore the epidemiological features of PMI in Vietnam and evaluate the burden on the national economy, readmission risk, and LOS using data collected from the Vietnam Social Insurance database.

## Materials and Methods

### Data Source

Our study analyzed data exported from a standardized national system, which is the electronic payment portal database under the management of the Vietnam Social Insurance (VSI) agency using an Oracle^©^ database. The system was implemented nationwide on January 1, 2017, and required all medical data from domestic hospitals to be entered and uploaded onto the VSI server to enable reimbursement (25). Data on care for all administrative levels and medical procedures are reported through the VSI database. The administrative levels in Vietnam are organized hierarchically from primary to tertiary care, while medical procedures include preventive and treatment care, medical consultations, maternity care, recovery, and prescribed medications. The 10th revision of International Classification of Diseases (ICD-10) codes was used to code VSI diagnosis data, while a domestic coding system adapted from the International Classification of Diseases, 9th Revision, Clinical Modification (ICD-9-CM) classification system is used for medical procedures (26). The data for this study were exported from January 1, 2017, to September 30, 2018.

### Study Participants

Patients aged 18 years old or above who had undergone at least one of seven procedures were eligible for the study: (1) spinal-neurological, (2) cardiothoracic, (3) vascular, (4) gastrointestinal, (5) urological, (6) orthopedic, and (7) plastic. Patients were excluded from the study if they had undergone another type of surgery within 30 days before being recruited into the study.

### Measurements

#### Myocardial Infarction

MI describes a clinical phenomenon where the bloodstream that supplies oxygen to cardiac muscle tissues is interrupted, with a consequent risk of myocardial ischemia and necrosis (1, 2). One of the most common reasons for myocardial infarction is a thrombosis that forms gradually and asymptomatically inside the coronary arteries (1, 2). Myocardial infarction is diagnosed through clinical symptoms of myocardial ischemia, electrocardiogram (ECG), the presence of relevant biomarkers, and imaging.

MI is diagnosed when the evidence of myocardial necrosis is available accompanied with clinical evidence of myocardial ischemia and the increase and/or decrease of cardiac biomarkers (troponin) with at least one value exceeding the 99th percentile upper reference limit, followed by one of the following criteria as follows (1, 3): (1) symptoms of ischemia; (2) changes in ECG indicating new ischemia as new changes of ST segment and T wave and left bundle branch block; (3) development of pathological Q wave recorded in ECG; (4) evidence of new loss of viable myocardium or regional wall motion abnormality detected by imaging; (5) evidence of thrombosis detected by coronary angiography or autopsy.

#### Readmission

We examined the impact of PMI on the readmission of surgical patients. Readmission refers to any overnight stay at a hospital. Readmission was assessed within 30 and 90 days after surgery.

#### Cost

A bottom-up approach was used to capture the total amount of cost reimbursed by VSI and any costs incurred by the patient that were ineligible for reimbursement by VSI. In these cases, direct medical costs were directly assessed. Direct medical costs were separated into three indicators, the total index treatment costs, costs post-operatively within 30 days of surgery, and costs within 90 days. The total index treatment cost is the sum of costs that patients paid for full episodes of initial treatment, while post-operative costs are the total index treatment cost and all extra costs incurred with 30 and 90 days after surgery. A full episode of initial treatment was considered as all stages that patients had completed during their hospital visit, from admission to discharge, and the corresponding costs relate to registration, consolation, testing, medications, and hospitalization. We calculated all the costs in USD with the 2018 rate at 1 USD = 23,255 Vietnamese dong.

#### Length of Stay

LOS is defined as the number of days of treatment during the index hospital admission, from the date of surgery to discharge. LOS at 30 and 90 days included both the stay for the index hospital admission and stays for any corresponding readmission.

### Statistical Analysis

To detect differences in patients' characteristics between the two groups (with and without PMI), we used Chi-squared tests for categorical variables and *t*-tests for continuous variables.

#### Effects of PMI on Readmission

Multivariable logistic regressions were carried out to evaluate the effect of PMI on readmission within 30 and 90 days after surgery. The models were adjusted for patient socio-demographic, emergency hospitalization status, and pre-operative comorbidities.

#### Matching

The propensity score matching (PSM) method was used with a matched ratio of one-to-one using nearest neighbor algorithm, to eliminate the risk of potential bias in PMI-related cost estimation as a result of distinction in socio-demographic and hospital characteristics between the two groups of participants. Each individual was assigned a propensity score, which was the output of the logistic regression. Subsequently, an individual in the first group was matched with another individual with the same propensity score in a second group for the purpose of promoting the characteristic balance in the analytic sample (27, 28). The covariates for the logistic regression included socio-demographic characteristics, hospital information, emergency status, and a list of chronic comorbidities provided by Elixhauser et al. (29).

#### Incremental Cost Estimation

We excluded patients with multiple complications to avoid the compounding effect and to achieve a more precise estimate of the burden attributable to PMI. We used the matched sample to estimate the incremental cost of PMI and LOS. A generalized linear model with a log link function was used for all the models. The Gamma distribution was chosen to reflect the right-skewed nature of the costs, while Poisson distribution was used to estimate the incremental LOS, reflecting the nature of the count data. The 95% confidence intervals (95% CI) were estimated based on the bootstrapping method with 1,000 replications.

A significance level of 0.05 was used for all statistical tests. Data were managed and analyzed using Stata v16 (StataCorp, College Station, TX, USA).

### Ethical Approval

All procedures performed in this study were in accordance with the ethical standards of the Ethical Review Board of Hanoi Medical University (IRB approval No. 67/HDDDDHYHN; Dated: March 24, 2017). All patient information was anonymous.

## Results

### Study Sample

**Figure 1:**
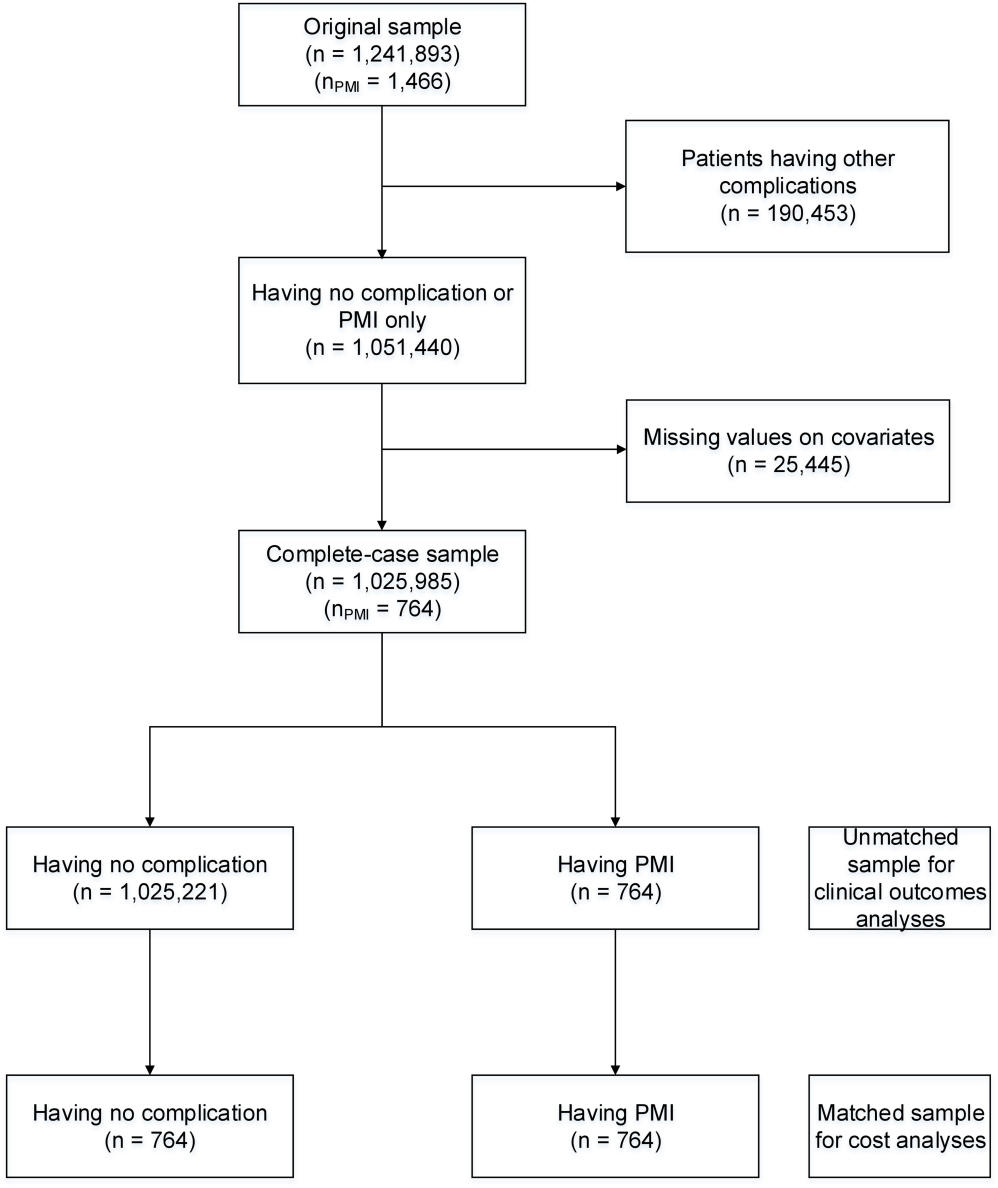
Flow diagram of study sample size. PMI, post-operative myocardial infarction.

Figure 1 describes the sample size of our study. The original study sample was 1,241,893 surgical patients, of which 1,466 patients had PMI (0.12%). After removing 190,453 patients with other post-operative complications and 25,445 samples with missing values for covariates, the complete case sample included 1,025,985 patients. In the matched sample for costs analysis, 764 patients with PMI were matched with 764 patients without PMI.

### Incidence of PMI

**Figure 2:**
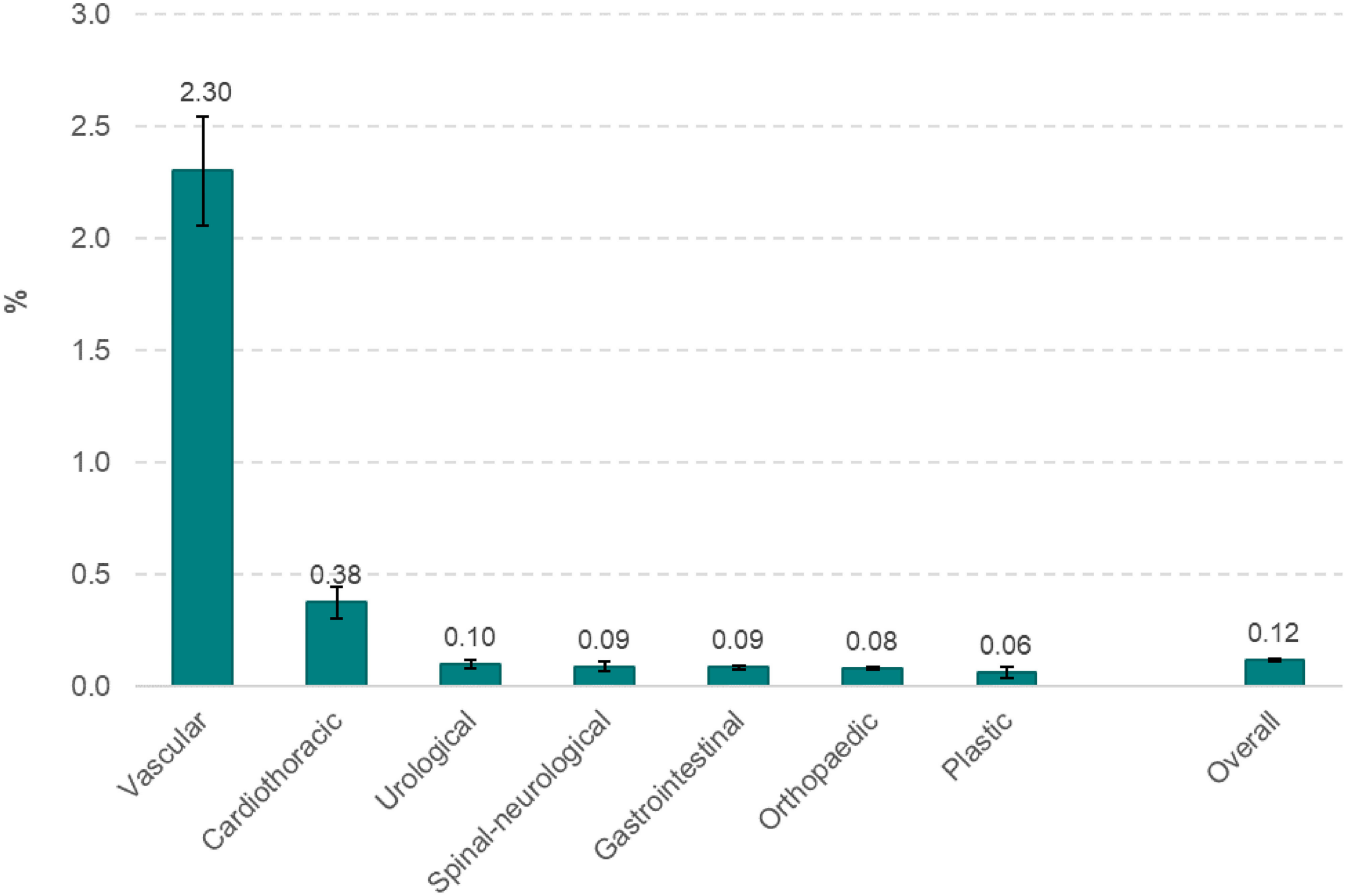
Incidence of post-operative myocardial infarction by seven types of surgery.

The incidence of PMI for each of seven types of surgery is shown in Figure 2. The incidence of PMI was highest with vascular surgery (2.30%), followed by cardiothoracic surgeries (0.38%). PMI was <0.1% in other types of surgery.

### Patient Characteristics

Table 1 summarizes the pre-operative characteristics of surgical patients with PMI before and after matching. In the unmatched sample, the patient's characteristics were significantly different between groups of patients with PMI (*n* = 764) and without PMI (*n* = 1,025,221). After matching 764 patients who had PMI with 764 patients with no post-operative complications using the PSM method, the distribution of patient characteristics was similar in the two groups and no significant differences were found.

**Table 1:** Baseline characteristics of surgical patients by myocardial infarction before and after matching.

**Factors**	**No PMI**		**PMI *n* (%)**	***P*** **-value**	
	**Unmatched sample *n* (%)**	**Matched sample *n* (%)**		**Unmatched sample**	**Matched sample**
**N**	1,025,221	764	764		
**Age**, mean (SD)	46.9 (17.3)	65.4 (16.2)	65.0 (15.9)	<0.001	0.60
**Gender**					
Male	613,861 (59.9)	457 (59.8)	462 (60.5)	0.74	0.79
Female	411,360 (40.1)	307 (40.2)	302 (39.5)		
**Region**					
Northern Midlands and Mountainous	134,976 (13.2)	49 (6.4)	50 (6.5)	<0.001	0.99
Red River Delta	229,401 (22.4)	169 (22.1)	170 (22.3)		
North Central and South Central Coast	259,894 (25.4)	136 (17.8)	129 (16.9)		
Central Highland	60,444 (5.9)	21 (2.7)	23 (3.0)		
Southeast	182,402 (17.8)	213 (27.9)	207 (27.1)		
Mekong River Delta	158,104 (15.4)	176 (23.0)	185 (24.2)		
**Emergency hospitalization**	196,872 (19.2)	217 (28.4)	207 (27.1)	<0.001	0.57
**Pre-operative concomitant diseases**					
Heart failure	7,055 (0.7)	44 (5.8)	49 (6.4)	<0.001	0.59
Valvular heart disease	3,940 (0.4)	9 (1.2)	9 (1.2)	<0.001	1.00
Peripheral vascular disease	2,109 (0.2)	8 (1.0)	10 (1.3)	<0.001	0.64
Hypertension	9,1907 (9.0)	252 (33.0)	252 (33.0)	<0.001	1.00
Paralysis	986 (0.1)	2 (0.3)	1 (0.1)	0.76	0.56
Chronic lung disease	15,907 (1.6)	32 (4.2)	28 (3.7)	<0.001	0.60
Diabetes	36,439 (3.6)	119 (15.6)	124 (16.2)	<0.001	0.73
Complicated diabetes	583 (0.1)	1 (0.1)	3 (0.4)	<0.001	0.32
Hypothyroidism	1,231 (0.1)	2 (0.3)	2 (0.3)	0.26	1.00
Chronic renal failure	6,069 (0.6)	20 (2.6)	27 (3.5)	<0.001	0.30
Liver diseases	24,108 (2.4)	13 (1.7)	16 (2.1)	0.64	0.57
Metastatic cancer	4,797 (0.5)	2 (0.3)	2 (0.3)	0.40	1.00
Cancer	44,609 (4.4)	24 (3.1)	21 (2.7)	0.030	0.65
Joint disease	11,616 (1.1)	6 (0.8)	7 (0.9)	0.57	0.78
Weight loss	8,453 (0.8)	18 (2.4)	18 (2.4)	<0.001	1.00
Fluid and electrolyte disorders	1,476 (0.1)	2 (0.3)	2 (0.3)	0.39	1.00
Anemia	3,483 (0.3)	2 (0.3)	2 (0.3)	0.71	1.00
Depression/addiction	585 (0.1)	0 (0.0)	0 (0.0)	0.51	–

*PMI, post-operative myocardial infarction; SD, standard deviation*.

### Impact of PMI on Readmission

Table 2 shows the results of multivariable logistic regression for the impact of PMI on readmission within 30 and 90 days. After controlling for socio-demographic, emergency hospitalization status, and pre-operative comorbidities, patients with PMI had higher odds of readmission within 30 and 90 days as compared to those who did not have a post-operative complication (OR = 3.45; 95%CI: 2.92–4.08; and OR = 4.39; 95%CI: 3.78–5.10, respectively).

**Table 2:** Impact of post-operative myocardial infarction on readmission within 30 and 90 days of surgical patients.

**Factors**	**30-day readmission**		**90-day readmission**	
	**OR**	**95%CI**	**OR**	**95%CI**
**PMI**	3.45^***^	2.92–4.08	4.39^***^	3.78–5.10
**Age**	1.01^***^	1.01–1.01	1.02^***^	1.01–1.02
**Gender** (Ref: Male)				
Female	0.85^***^	0.84–0.86	0.89^***^	0.88–0.90
**Region** (Ref: Northern Midlands and Mountainous)				
Red River Delta	0.86^***^	0.84–0.88	0.85^***^	0.83–0.87
North Central and South Central Coast	0.82^***^	0.80–0.84	0.86^***^	0.84–0.87
Central Highland	0.83^***^	0.80–0.86	0.84^***^	0.82–0.87
Southeast	0.60^***^	0.58–0.61	0.60^***^	0.59–0.61
Mekong River Delta	0.64^***^	0.62–0.66	0.63^***^	0.61–0.65
**Emergency hospitalization**	1.12^***^	1.10–1.14	1.09^***^	1.07–1.11
**Hospital classification** (Ref: Level 2 or lower)				
**Pre-operative concomitant disease**				
Heart failure	1.03	0.95–1.11	1.02	0.95–1.09
Valvular heart disease	1.17^**^	1.06–1.30	1.16^**^	1.06–1.28
Peripheral vascular disease	1.27^***^	1.11–1.44	1.18^**^	1.05–1.33
Hypertension	1.13^***^	1.10–1.16	1.14^***^	1.12–1.17
Paralysis	2.13^***^	1.81–2.51	1.97^***^	1.69–2.30
Chronic lung disease	1.22^***^	1.16–1.28	1.29^***^	1.24–1.35
Diabetes	1.22^***^	1.18–1.26	1.23^***^	1.19–1.26
Complicated diabetes	1.11	0.88–1.42	1.16	0.94–1.44
Hypothyroidism	0.97	0.80–1.17	1.04	0.89–1.23
Chronic renal failure	3.27^***^	3.08–3.48	3.09^***^	2.91–3.27
Liver disease	1.07^***^	1.03–1.12	1.08^***^	1.05–1.13
Metastatic cancer	2.35^***^	2.19–2.53	2.26^***^	2.11–2.42
Cancer	5.52^***^	5.40–5.65	5.11^***^	5.00–5.22
Joint disease	0.95	0.89–1.01	0.99	0.93–1.04
Weight loss	1.07^*^	1.00–1.15	1.04	0.98–1.11
Fluid and electrolyte disorders	1.13	0.97–1.32	1.10	0.95–1.26
Anemia	1.42^***^	1.29–1.56	1.43^***^	1.31–1.55
Depression/addiction	1.30	0.98–1.70	1.21	0.94–1.55

*All estimates were calculated using unmatched sample*,

*** *p < 0.001;*

** *p < 0.01;*

* *p < 0.05*.

*OR, odds ratio; 95%CI, 95% confidence interval; PMI, post-operative myocardial infarction; Ref, reference*.

### Incremental Cost and LOS Due to PMI

The incremental costs and LOS due to PMI are shown in Table 3. A significant increase was found in most types of cost, except for the cost of outpatient visits and the drug cost associated with outpatient visits. Specifically, the incremental cost associated with PMI of the index treatment was 2,176.7 USD (95% CI: 1902.7–2450.8). The increments of total costs within 30 days after surgery were 4,490.9 USD (95% CI: 3882.3–5099.5) and within 90 days were 4,724.6 USD (95% CI: 4111.5–5337.8), which is equivalent to 175% and 184% of the GDP per capita in Vietnam in 2018, respectively. The service cost related to readmission contributed the largest part of the total costs.

**Table 3:** Incremental costs and length of hospital stay due to post-operative myocardial infarction complication.

	**Mean cost (USD)**		**Incremental cost of PMI,** **USD (% GDP^a^)**	**95% Bootstrap** **CI**	***P*-value**
	**No PMI**	**PMI**			
**N**	764	764			
**Treatment cost**					
Total cost of the index treatment	1019.9	3196.6	2176.7 (84.8)	1902.7–2450.8	<0.001
**30-day costs**					
Service cost for 30-day readmission	858.8	2773.8	1914.9 (74.6)	1649.0–2180.8	<0.001
Drug cost for 30-day readmission	177.4	547.5	370.2 (14.4)	292.6–447.7	<0.001
Service cost for 30-day outpatient visits	79.8	89.7	9.9 (0.4)	−19.7–39.5	0.511
Drug cost for 30-day outpatient visits	37.6	56.8	19.2 (0.7)	−0.8–39.1	0.060
Total 30-day cost	2173.4	6664.3	4490.9 (175.0)	3882.3–5099.5	<0.001
**90-day costs**					
Service cost for 90-day readmission	882.7	2982.3	2099.6 (81.8)	1834.4–2364.8	<0.001
Drug cost for 90-day readmission	187.9	572.3	384.4 (15.0)	305.4–463.5	<0.001
Service cost for 90-day outpatient visits	96.6	126.9	30.3 (1.2)	−2.9–63.5	0.074
Drug cost for 90-day outpatient visits	49.7	83.3	33.6 (1.3)	10.9–56.3	0.004
Total 90-day cost	2236.7	6961.4	4724.6 (184.1)	4111.5–5337.8	<0.001
	**Difference in LOS**				
**Length of stay (days)**					
Length of hospital stay of the index treatment	11.8	15.3	3.5	2.4–4.6	<0.001
Total length of treatment within 30 days^b^	13.0	17.9	4.9	3.6–6.2	<0.001
Total length of treatment within 90 days^c^	14.0	19.7	5.7	4.2–7.2	<0.001

a *Compared to GDP per capita in Vietnam in 2018 (2,566 USD)*.

b *Includes length of hospital stay in the treatment and 30-day re-hospitalization periods*.

c *Includes length of hospital stay in the treatment and 90-day re-hospitalization periods. All estimates were calculated using matched sample*.

*CI, confidence interval; GDP, gross domestic product; LOS, length of stay; PMI, post-operative myocardial infarction; Ref, reference*.

LOS was also higher in patients with PMI, with an average increase of 3.5 days for the index treatment, 4.9 days within 30 days, and 5.7 days within 90 days after surgery. All the increments in LOS were significant with a *p*-value < 0.001.

## Discussion

Cardiovascular complications have become a significant public health issue in recent years. Alongside putting persons at risk of poor prognosis, PMI exposes patients to invasive treatment and diminishes their post-operative quality of life. Any poor outcome due to cardiovascular complications, especially PMI, increases the relevant medical costs, and so generates an economic burden that negatively affects patients, especially those living in LMICs. Nevertheless, understanding this impact requires a great deal of effort on data collection at the national level. For these reasons, this present study set out to describe the epidemiological characteristics of PMI in Vietnam and estimate PMI-related medical cost at the individual level, as well as the correlation between PMI and readmission. The data analyzed in our study came from a standardized national system under the management of the VSI.

We found that 1,466 (0.12%) participants were diagnosed with PMI in the initial sample. PMI predominantly occurred after vascular surgery, with 2.3% of patients, and was less frequently recorded among the other types of surgery. Patients with PMI were shown to have higher odds of 30- and 90-day readmission compared to patients without PMI. The total costs of the PMI-related treatment were higher in the PMI group than in the non-PMI groups, and the same trend was found for LOS days. The estimated incremental cost was US$2,176.70 for the index treatment, which accounted for 84.8% of GDP per capita in the contemporary year.

The incidence of PMI in our study was calculated at 0.12%, which is lower than the range of incidence of 0.3–33.3% reported in the literature review by Gualandro et al. (9). The figure of Poldermans et al. showed that the rate of PMI occurring within 30 days after surgery was up to 32.7%; however, the sample size was only 101 patients with extensive ischemia (5), compared to more than one million surgical patients in our study. It should be noted that the incidence might vary across studies due to differences in either socio-demographic or clinical characteristics or disease definitions (9). Kertai et al. found a PMI incidence of 8.9% among patients experiencing abdominal aortic aneurysm surgery (30), while Durazzo et al. found a rate of 11% for patients undergoing arterial surgery (31), which is higher than the rate of 2.3% in our study. However, the sample size of the two studies was significantly smaller than the VSI sample and focused on specific groups.

We found that participants with PMI had an adjusted OR of 30-day readmission 3.45-times higher than ones without PMI, and the effect was even stronger for 90-day readmission. Smilowitz et al. revealed that, in 2014, patients in the US diagnosed with perioperative acute MI were more likely to undergo readmission within 30 days after discharge (15). The finding by Smilowitz is consistent with our results, and the analysis of Smilowitz was based on a national database (15). The same trend was displayed in a systematic review of 14 studies on PMI topics and MI was recorded as a potential risk factor for 30-day readmission with the pool OR at 2.26 (14).

Although national social care packages have been widely developed in the attempt to support patients in reducing health-related burdens, the accumulative medical care contributes to a significant expense for patients. Furthermore, even though medical costs are partly reimbursed by VSI, the high level of cost may prevent PMI patients from seeking treatment, leading to a higher risk of poor prognosis due to the urgent nature of the disease. We found that patients with PMI had to pay three times more than patients without PMI. As a result, the incremental cost equated to roughly 84.8% of the 2018 year GDP per capita. This high impact is in line with results published in previous studies (20, 32, 33). Nguyen et al. showed that acute MI treatment costed Vietnamese patients US$ 2,503 per hospitalization in 2013, which is lower than our estimated cost in 2018 (20). This difference may be because our sample size was larger than the sample of only 89 patients analyzed by Nguyen (20). In addition, we collected data from the VSI database that contains medical cost information across regions in Vietnam, while the data range recorded by Nguyen focused on one regional hospital (20). The variation in currency exchange rates across years may also contribute to the differences between the two studies. Our finding was equivalent to that of Soekhlal et al. who found a mean cost of acute MI treatment of US$3,094 for 2012 in Netherlands (32). The data in a study by Soekhlal was collected from nationwide at the same administrative levels as our database (32). In 2005, Tiemann et al. calculated total acute MI-related cost per case was ~US$3,027 using a European database (34). Moreover, the estimated cost increased across European countries based on the availability of modern invasive procedures (34). The differences in treatment cost remained unchanged between two groups after summing all the costs incurring within 30 and 90 days after surgery. In addition, PMI generated the risk of suffering a longer hospital LOS. This negative point is reflected in the values of the PMI group; which were significantly higher than the non-PMI group for both the index treatment and post-operative duration.

Our study applied a standardized national system under VSI management that provided the individual data across all regions in Vietnam. The large scale of the database strengthened the costing analysis that was central to this study. The propensity scores matching was executed to reduce the imbalance in to characteristics between two groups. Hence, the estimated costs were adjusted for the socio-demographic characteristics, hospital information, emergency status, and history of comorbidities.

There were limitations to our study. The analysis did not include indirect costs that possibly exist as a result of the negative effects of PMI treatment. The indirect costs may emerge due to the expenses of either additional functional foods or drugs outside regular hospital medications. Also, the VSI database does not cover the data on care for cases seeking treatment in the private sector. The estimated medical costs are not adjusted for the different thresholds of reimbursement that align with VSI regulations and are not stratified according to different types of surgery. The retrospective data potentially contains a certain number of missing records as well as the issues of data uncertainty, which could potentially affect the precision of the estimates.

## Conclusion

Even though the incidence of PMI has decreased in recent years, medical costs affect the lives of patients and create a heavy burden on the national economy. The other impact of PMI on LOS and risk of readmission, if not properly considered, harm the patient's quality of life and prompt the need for medical care. Additional research is needed to explore the economic burdens of PMI on individual and national levels across various types of surgery and social status and to estimate the medical costs relating to the private sector.

## Data Availability Statement

The raw data supporting the conclusions of this article will be made available by the authors, without undue reservation.

## Author Contributions

MB, QK, and PD conceived the study, performed the official statistical analyses, interpreted the results, and wrote the manuscript. MB and TT collected and cleaned data. CL, TN, BT, DD, TD, TT, HP, XD, and QL provided the critical revision of the manuscript for important intellectual content. All authors have read and approved the final manuscript.

## Conflict of Interest

The authors declare that the research was conducted in the absence of any commercial or financial relationships that could be construed as a potential conflict of interest.

## Publisher's Note

All claims expressed in this article are solely those of the authors and do not necessarily represent those of their affiliated organizations, or those of the publisher, the editors and the reviewers. Any product that may be evaluated in this article, or claim that may be made by its manufacturer, is not guaranteed or endorsed by the publisher.
